# Clinical, Molecular and Genetic Characteristics of Early Onset Gastric Cancer: Analysis of a Large Multicenter Study

**DOI:** 10.3390/cancers13133132

**Published:** 2021-06-23

**Authors:** Anna Pocurull, Cristina Herrera-Pariente, Sabela Carballal, Joan Llach, Ariadna Sánchez, Laura Carot, Josep María Botargues, Miriam Cuatrecasas, Teresa Ocaña, Francesc Balaguer, Luis Bujanda, Leticia Moreira

**Affiliations:** 1 Gastroenterology Department, Hospital Clínic de Barcelona, Centro de Investigación Biomédica en Red de Enfermedades Hepáticas y Digestivas (CIBERehd), Institut d’Investigacions Biomediques August Pi i Sunyer (IDIBAPS), Universitat de Barcelona, 08036 Barcelona, Spain; pocurull@clinic.cat (A.P.); cristina.herrera@ciberehd.org (C.H.-P.); carballal@clinic.cat (S.C.); jllachr@clinic.cat (J.L.); asanchezg@clinic.cat (A.S.); mocana@clinic.cat (T.O.); fprunes@clinic.cat (F.B.); 2 Gastroenterology Department, Hospital del Mar, 08003 Barcelona, Spain; lcarot@psmar.cat; 3 Gastroenterology Department, Hospital Universitari de Bellvitge, 08097 Barcelona, Spain; jbotargues@bellvitgehospital.cat; 4 Department Pathology, Hospital Clínic de Barcelona, Centro de Investigación Biomédica en Red de Enfermedades Hepáticas y Digestivas (CIBERehd), Institut d’Investigacions Biomediques August Pi i Sunyer (IDIBAPS), Universitat de Barcelona, 08036 Barcelona, Spain; mcuatrec@clinic.cat; 5 Gastroenterology Department, Biodonostia Health Research Institute, Centro de Investigación Biomédica en Red de Enfermedades Hepáticas y Digestivas (CIBERehd), Universidad del País Vasco (UPV/EHU), 20014 San Sebastián, Spain; LUIS.BUJANDAFERNANDEZDEPIEROLA@osakidetza.eus

**Keywords:** gastric cancer, early onset cancer, DNA mismatch repair, hereditary cancer, familial cancer

## Abstract

**Simple Summary:**

Gastric cancer is one of the most common cancers worldwide, showing high mortality rates. A small portion of gastric cancer patients, known as early onset gastric cancer (EOGC) patients, develop the disease before age 50, and their characteristics are poorly described. Thus, our main objective was to describe the clinical, molecular, and genetic characteristics of EOGC in a large multicenter cohort of patients. We were able to identify that most EOGC cases have similar characteristics: diagnosed at advanced stage, diffuse type, and infrequent DNA mismatch repair somatic deficiency. Although familial aggregation of gastric cancer was uncommon, a germline genetic mutation was identified in 25% of the patients tested. Our results show that EOGC has a marked genetic heterogeneity. Thus, it is essential to consider familial history of tumors, not only GC, in order to select adequate patients to perform a suitable genetic counseling and enhance the emerging use of multigene panels.

**Abstract:**

Gastric adenocarcinoma (GC) is a common tumor with high morbidity and mortality. Only 7% of patients with GC are diagnosed before age 50 (early onset gastric cancer (EOGC)), and their characteristics have been poorly described. We aimed to describe clinical, molecular, and genetic characteristics of EOGC. A total of 309 patients with EOGC were retrospectively studied in four Spanish centers. Personal information, family history, and tumor information were registered. Germinal genetic analysis was performed in patients who met current criteria of a hereditary syndrome at the time of diagnosis. The median age at diagnosis was 44 years. The majority (73.3%) of tumors were diffuse, and 78.3% were diagnosed in an advanced stage. Familial aggregation of GC was present in 18/117 (15.4%) cases, and 5/117 (4.3%) met criteria for familial GC. MMR-IHC was performed in 126/309 (40.7%) tumors: 4/126 (3.1%) had loss of expression in MLH1/PMS2, without an associated germline mutation. Sixteen germline genetic analyses were performed, detecting a pathogenic variant in four (25%) cases: one in *BRCA2*, one in *TP53*, and two in *CDH1*. Most EOGC are diffuse and diagnosed in an advanced stage. In these patients, DNA MMR system deficiency is uncommon. Although familial aggregation was observed in only 15% of cases, a germline mutation was found in 25% of patients tested with clinical criteria. This demonstrates that EOGC has a marked genetic heterogeneity, reinforcing the importance of an accurate genetic counseling and enhancing the emerging use of multigene panels.

## 1. Introduction

Gastric cancer (GC) is the fifth most common and the third most deadly cancer in the world [1], representing a worldwide health problem [2]. The average age at diagnosis is 60 years, only 7% occur before age 50 and 2% before age 40 [3]. The etiology of GC is multifactorial, with *Helicobacter pylori*, diet factors, and tobacco being the main environmental agents implicated in its pathogenesis [4]. Although most GC cases are sporadic, a familial aggregation is observed in approximately 10% of cases, with an underlying genetic cause identified in up to 5% of all GC [5]. Familial characteristics that suggest a hereditary predisposition include the existence of several affected family members, an autosomal dominant pattern of inheritance, disease presentation at young ages, and association with other extra-gastric neoplasms [6]. 

In terms of these assumptions, there are mainly three clinical situations where familial predisposition to GC may be found [7]. First, hereditary syndromes with higher risk for GC, including two entities: gastric adenocarcinoma and proximal polyposis of the stomach (GAPPS) [8], and the most common inherited GC syndrome, hereditary diffuse gastric cancer (HDGC). It is characterized by two or more cases of GC at any age in first or second relatives, with at least one confirmed diffuse gastric cancer (DGC); or personal history of DGC before the age of 50; or personal or family history (first- or second-degree relatives) of DGC and lobular breast cancer, with one being diagnosed before 70 years [9]. This syndrome is mainly caused by *CDH1* germline mutations, which encode the tumor suppressor protein E-cadherin. However, during the last years, another gene, *CTNNA1*, has also been identified in HDGC families [10]. 

Other clinical situations are hereditary syndromes with higher risk for GC and other tumors, including Lynch syndrome and, less commonly, familial adenomatous polyposis (FAP), Peutz–Jeghers syndrome, juvenile polyposis, Li–Fraumeni syndrome, and Cowden syndrome [11], with germline mutations in different genes. The last clinical situation is familial intestinal gastric cancer (FIGC) characterized by familial aggregation of intestinal GCs without an identified inherited cause. FIGC is defined as two or more cases of GC in first-degree (FDR) or second-degree relatives (SDR), with at least one confirmed case of intestinal histology in someone younger than 50 years, or three or more confirmed cases of intestinal GC in FDR or SDR, regardless of age [12,13]. 

In spite of this, the genetic cause is not identified in a high percentage of GC patients [14,15]. This fact is especially important in early-onset gastric cancer cases (EOGC) because 90% of these young patients do not have a family history, hampering identification and early diagnosis [16]. The remaining 10% of EOGC cases that have a family history are explained by the previously mentioned hereditary syndromes.

The definition of early onset gastric cancer (EOGC) varies across studies, but one of the most accepted definitions includes those diagnosed at the age of 50 or younger. Although the incidence of GC is declining globally, EOGC is increasing [17]. In fact, a recent study has reported that nowadays EOGC comprises up to 30% of all cases of GC in the United States [18]. EOGC has been associated with some clinical and pathological characteristics, such as predomination of diffuse histology and infrequent association with intestinal metaplasia [19,20]. Moreover, EOGC is usually diagnosed in an advanced stage, associated with a high mortality. However, clinical and molecular features of EOGC have been poorly described [16]. 

Identification of individuals at high risk of GC allows us to establish preventive measures, early diagnosis, and personalized treatments; thus, we aimed to describe the clinical, molecular, and genetic characteristics of EOGC (≤50 years) in order to identify high-risk forms of GC. 

## 2. Materials and Methods

### 2.1. Study Population

Patients with GC diagnosed before 51 years old were retrospectively studied at four centers in Spain between 1999 and 2018. The study was approved by the Institutional Review Board (or Ethics Committee) of Hospital Clínic in Barcelona (register number 2015/0153, date of approval 22/04/2015).

Clinical and demographic data were evaluated through electronic clinical reports, including environmental risks factors such as tobacco consumption, alcohol intake, and *Helicobacter pylori* infection. 

Personal and family history of GC and other tumors related with hereditary syndromes (i.e., HDGC, Peutz–Jeghers syndrome, Lynch syndrome, familial adenomatous polyposis, HBOC, juvenile polyposis, and Li–Fraumeni syndrome) were registered (including FDRs and SDRs). Patients who met criteria of familial GC were also identified.

### 2.2. Tumor Characteristics

The tumor characteristics were analyzed by histological report. The location, diagnostic stage (TNM), histologic features (intestinal, diffuse, or mixed), and grade of differentiation were considered. 

Tumor mismatch repair (MMR) deficiency was evaluated by immunostaining including analysis of MLH1, MSH2, MSH6, and PMS2 protein expression, as previously described [21].

### 2.3. Germline Genetic Analysis

Germline genetic testing was performed on genomic DNA isolated from peripheral blood leukocytes by both multiple ligation probe amplification analysis and direct sequencing. The analysis was performed through a commercial multigene panel (Trusight Cancer v1, Illumina Inc., San Diego, CA, USA) involving the most frequent genes related to GC germline predisposition (*MLH1*, *MSH2*, *MSH6*, *PMS2*, *CDH1*, *EPCAM*, *BRCA1*, *BRCA2*, *PALB2*, *TP53*, *APC*, *MUTYH*, *STK11*, *SMAD4*/*BMPR1A*, *PTEN*). 

The genetic test was performed in patients with available germline DNA who fulfilled the diagnostic criteria of a hereditary syndrome related to GC at the time of diagnosis or in whom the tumoral analysis of DNA mismatch repair proteins was altered (i.e., loss of protein expression of MLH1, MSH2, MSH6, or PMS2) [13].

### 2.4. Statistical Analysis

All data were analyzed using the 22.0 SPSS software package (IBM SPSS Statistics for Window, Version 22.0. Armonk, NY: IBM Corp.). 

Baseline characteristics were described in percentages for categorical data, using median, range, and interquartile range (IQR). When information was missing, the denominator was accordingly to patients with available data. Univariate binary logistic regression was performed for selection of variables associated with the diagnosis of a hereditary cancer. For multivariable logistic regression analyses, only candidate variables with *p*-values of ≤0.05 on univariate analysis were used in the final multivariate model. Odds ratios (ORs) with 95% confidence intervals (CIs) were included to quantify the magnitude of the association.

## 3. Results

### 3.1. General Characteristics

Three hundred and nine patients with EOGC were included. Clinico-pathological features of patients included in the study are summarized in Table 1. 

The median age at diagnosis was 44 years old (IQR 40-48, range 33), with a predominance of men, with 191 (61.8%) cases. Related to environmental risk factors, 77/169 (45%) were smokers and 21/105 (20%) had chronic alcohol consumption, whereas in 24/82 (29%) cases, a *Helicobacter pylori* infection was detected. Most of the patients were from Spain; however, 8/309 (2.6%) were from South America, 4/309 (1.3%) were from Asia, and 4/309 (1.3%) were South African.

Out of the 309 (2.9%) patients, 9 had previously developed other tumors, including breast and/or ovarian in 5/309 (1.6%), lung in 1/309 (0.3%), cervix in 1/309 (0.3%), thyroid in 1/309 (0.3%), and Hodgkin lymphoma in 1/309 (0.3%). 

A total of 18/117 (15.4%) patients presented familial aggregation of GC (≥1 FDR or SDR affected), and 5/117 (4.3%) met criteria for FIGC. A total of 67/117 (57.2%) patients had family history of cancers related with a hereditary syndrome, mainly ovarian and/or breast in 15/117 (12.8%), followed by colorectal cancer in 8/117 (6.8%) cases and other types of tumors in 39/117 (34%). Detailed characteristics are described in Table 1. 

### 3.2. Tumor Characteristics

The predominant tumor histology was diffuse, observed in 118/161 (73.3%) of the cases, and the signet ring cell subtype was detected in 38/118 (32%). Among patients with diffuse GC and *H. pylori* information available, in 8/45 (17.8%), the infection was present. Among patients with intestinal GC subtype with *H. pylori* status available, 3/14 (21.4%) were infected. No statistically significant differences regarding *H. pylori* infection and histology subtype were found (*p* = 0.09). According to the WHO classification (2019), the degree of differentiation only applies to the intestinal GC, and thus within this subtype, 9/43 (39.1%) were poorly differentiated tumors. Regarding tumor location, the most common sites were the body in 111/203 (55%) and antrum in 50/203 (25%). An advanced stage (III/IV) at diagnosis was present in 166/212 (78.3%) cases, and only in 44/212 (20.8%) was the diagnosis at an early stage (I/II). 

In 122/205 (59.5%) cases, surgery with or without chemotherapy was performed; 67/205 (32.7%) patients were treated with chemotherapy (CT) +/− radiotherapy (RT) alone, and 16/205 (7.8%) patients did not receive a specific oncological treatment. 

**Figure 1:**
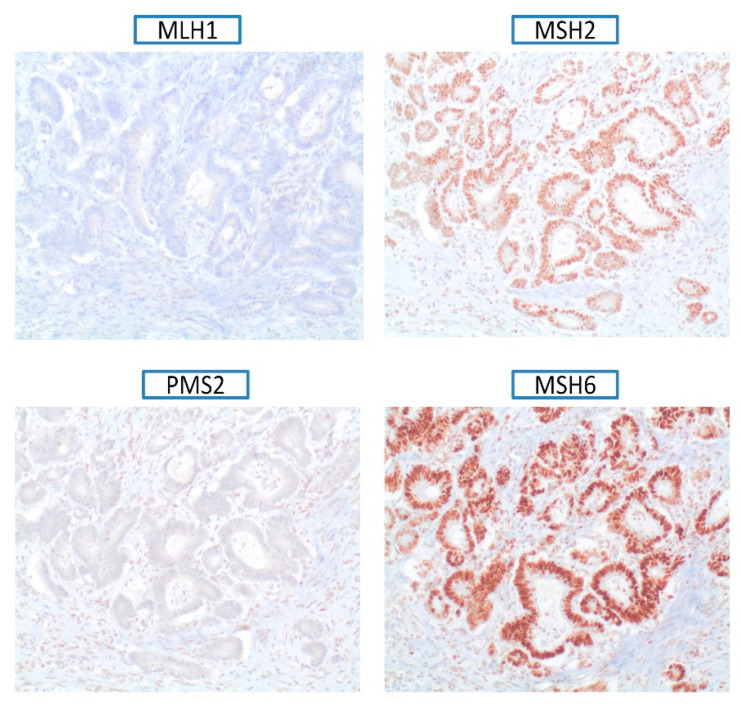
Immunohistochemistry of DNA mismatch repair proteins (MMR-IHC) in gastric cancer tissue loss of protein expression of MLH1 and PMS2, and normal protein expression of MSH2 and MSH6.

**Figure 2:**
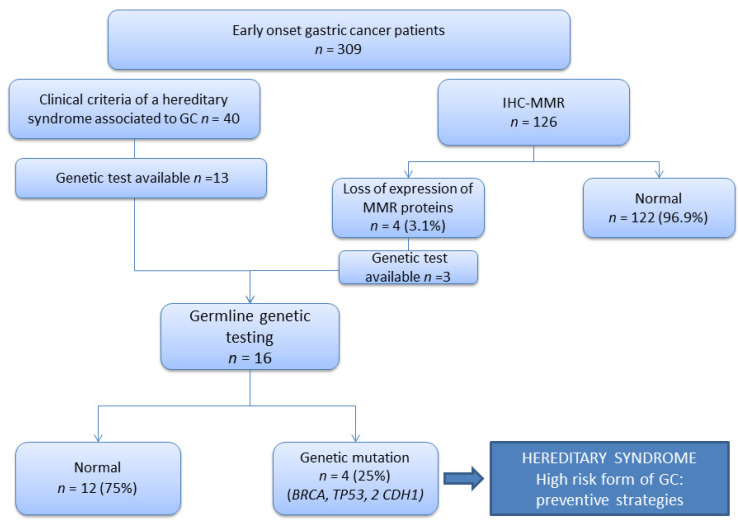
Scheme followed summarizing the results of IHC-MMR and germline genetic testing.

The immunohistochemistry of DNA mismatch repair proteins (MMR-IHC) was performed in 126 out of 309 (40.7%) tumors, and only 4/126 (3.1%) showed loss of protein expression, specifically MLH1/PMS2 (Figure 1). The tumor and MMR-IHC characteristics are shown in Table 2 and Figure 2, respectively. 

**Figure 3:**
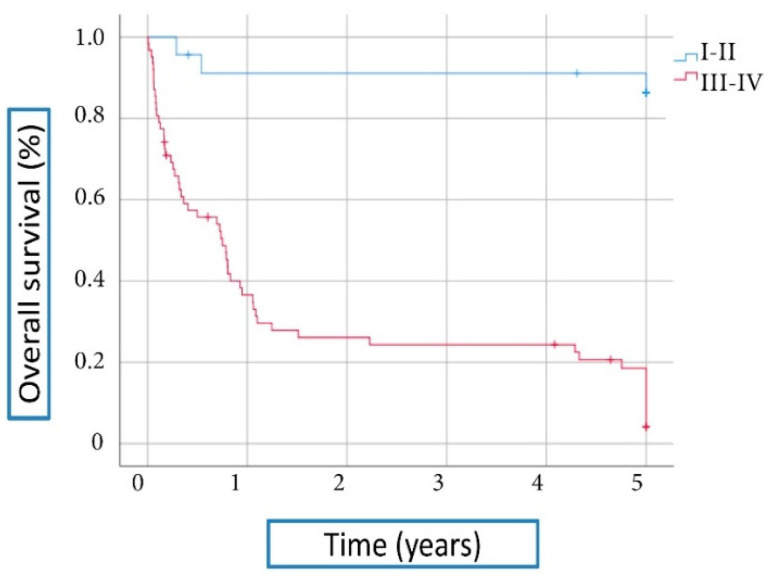
Overall survival rate based on diagnostic stage (stage I–II vs. stage III–IV). There is a significant difference in the 5-year survival rate: stage I–II 87% vs. stage III–IV 11.3%, *p* = 0.0001.

Regarding survival, with a median follow up of 7.6 years (IQR 17-38), a 5-year survival rate of 32.6% was observed, with a significant difference based on clinical stage (stage I–II 87% vs. stage III–IV 11.3%, *p* = 0.0001; Figure 3), and diffuse histology was associated with worse prognosis (*p* = 0.019); no differences in gender, age, family history of GC, *H. pylori* infection, smoking or alcohol consumption, tumor differentiation grade, or MMR-IHC were detected. 

### 3.3. Germline Genetic Analysis

Genetic analysis was performed in the 16 patients with available germline DNA out of the 44 patients that fulfilled clinical criteria of germline testing. Among them, in 11/16 (68.7%) patients, the analysis was performed due to the fulfillment of criteria for HDGC, in 2/16 patients because they met criteria of HBOC; and in 3/16 cases, the genetic analysis was performed on the basis of a somatic loss of expression of MLH1/PMS2 proteins at IHC. In the remaining 28/44 patients, the analysis was not performed because the DNA was not available.

A germline genetic mutation was identified in 4/16 (25%) cases, one at 49 years old (with personal history of GC and breast cancer) and another three with GC younger than 41 years old. The mutated genes detected were *BRCA2* and *TP53*, and in two cases, *CDH1* (Table 3). Integrative Genomics Viewer was used for visualization of these variants; Figure S1 shows an example of two of these variants. None of those patients had tumors with loss of expression in DNA mismatch repair protein (MMR), and only one of them reported family history of GC. Within four patients with an altered MMR-IHC, the germline genetic analysis performed in three patients did not identify any pathogenic variant, and in the remaining patient, the germline analysis could not be performed because he died before the MMR-IHC was done.

### 3.4. Factors Associated with High Risk of Gastric Cancer

Personal characteristics as well as family history were analyzed in order to identify risk factors of a hereditary GC syndrome (germline mutation identified). No statistically significant factor associated with the presence of a germline mutation was identified. However, family history of other neoplasms showed a trend towards statistical significance with *p* = 0.057. 

## 4. Discussion

It is well known that EOGC has clinical and pathological differences with older onset GC [22], although their clinical and molecular characteristics have been poorly reported [16]. In the present study, we describe clinical, molecular, and genetic characteristics of 309 EOGC patients. Our study shows that most of EOGC are histologically diffuse (73%, in comparison with 32% in older patients), poorly differentiated, and diagnosed at an advanced stage, supporting what has been previously described in other studies [18,19,20,23]. Moreover, as the already reported trend of increasing rate in general population [24] of proximal GC over the distal location, we observed in our study that more than half of the tumors were located in the gastric body. This could be explained not only due to the diffuse histology, but also because the low incidence of *Helicobacter pylori* infection (less than 30%) and a high proportion (almost 60%) of patients with positive oncological family history, suggesting a different carcinogenesis process. 

Although there are known characteristics of EOGC, there are some inconsistent data between studies, i.e., some studies reported a female predominance [23], while in others there was an increasing trend for males or without a significant difference between genders [25,26]. In the present study, a male predominance was identified. 

On the basis of different features, our study attempted to deepen in the clinical and molecular characterization of EOGC with the ultimate goal of being able to identify high-risk individuals and establish preventive measures, early diagnosis, and personalized treatments.

Lynch syndrome (LS), one of the most common cancer hereditary syndromes, carries a cumulative risk of GC of 11–19% [27,28]. However, DNA mismatch repair deficiency is exceptional in GC [29]. In order to consider the diagnoses of possible LS, we analyzed the MMR system deficiency, observing that loss of protein expression was an infrequent event, and only 4/126 (3.1%) patients displayed it. The low incidence of MMR deficiency in EOGC is probably related to the high proportion of diffuse tumors, wherein MSI is less common [30] and also related to the fact that genomically stable tumors are usually diagnosed at an earlier age [31]. Moreover, in cases with MMR deficiency, we did not find a correlation with a germline mutation, suggesting a somatic loss origin due to hypermethylation of the *MLH1* promoter gene [32]. Thus, based upon our results, in this subgroup of patients, systematically analysis of MMR deficiency to rule out Lynch syndrome through IHC is likely not very useful. 

Germline analysis was performed in 16 patients, representing 36% of patients who met clinical criteria for genetic testing according with the current guideline at that moment of the study (i.e., 2018–2019) [13]. Despite the fact that genetic testing could not be performed in the whole cohort of patients (due to loss of follow up or death), we found a germline mutation in 25% of tested patients. These mutations were located on *BRCA2*, *CDH1*, and *TP53* genes. Both patients with *CDH1* germline mutations displayed familial history of GC, while patients with *BRCA2* and *TP53* germline mutations showed familial history of ovarian and breast cancer, and breast and colorectal cancer, respectively. Thus, although familial aggregation of GC was present in only 15% of cases and the majority of patients with a germline mutation did not have familial aggregation of GC, family history of other tumors related with a hereditary syndrome was common. Therefore, this observation reinforces that, although family history of GC is poorly predictive, a very accurate medical personal and family history including any tumor type is mandatory in order to select the appropriate candidates for genetic testing. 

Analyzing other studies, Tedaldi et al. focused on 96 patients that fulfilled different criteria such as HDGC criteria, suspected Lynch syndrome, familial aggregation, or patients with polyps and family history of GC. They sequenced 94 genes involved in cancer predisposition, identifying eight different *CDH1* pathogenic/likely patogenic mutations in nine different patients with DGC and a mean age of almost 40 years. Although they identified more variants in other genes, their carriers were patients with more than 50 years old, and therefore they cannot be considered as an early onset cohort [33]. Moreover, a Canadian cohort was studied using single-site and multi-gene panels. The authors identified mutations in *CDH1* and *BRCA2* in five and two patients with DGC before the age of 50, respectively. Comparing with the present study, similar results were obtained, although the *TP53* gene was not identified in this cohort [34]. However, a study performed by Vogelaar et al. did not identify any mutation neither in *CDH1* nor in *CTNNA1* in a cohort of 54 GC EOGC patients [35].

The relevance of the diagnosis of a hereditary syndrome is the opportunity to establish prevention and early diagnosis measures. For example, specifically in the context of *CDH1* mutation carriers, most asymptomatic individuals do not have macroscopic lesions on endoscopic examinations; however, intramucosal foci of gastric cancer, usually multiple, are observed in the surgical specimens. Therefore, it is recommended that one perform prophylactic total gastrectomy in carriers of a pathogenic variant who are older than 20 years [36,37]. Annual endoscopic screening is reserved for individuals who do not accept prophylactic gastrectomy, patients with a variant of uncertain significance, and patients in whom the germline mutation has not been identified. This point is reflected in our cohort, wherein the two patients with a *CDH1* mutation were diagnosed at stage IV (metastatic). In one case, two relatives were carriers of the mutation, with normal upper gastroscopy but with multiple focuses of diffuse adenocarcinoma, both in early stages (T1a and T1b, N0, M0). In the second patient, no additional *CDH1* mutation carriers were identified.

This study has some limitations, most of them because the data obtained were collected retrospectively, which implies potential inclusion biases and the difficulty to obtain the information of some variables: Epstein–Barr virus infection; associated gastritis; HER-2 or PDL1 status; and, as mentioned previously, germline testing, which was only evaluated in 16/44 (36%) of patients who met clinical criteria for genetic testing, and *CTNNA1* was not performed. Moreover, it is important to mention that *H. pylori* status was available in only 82 cases, being positive in 24 of them (29%); however, no difference regarding *H. pylori* prevalence among diffuse and intestinal histology was detected. In this context, other studies have been focused on the role of *H. pylori* infection and EOGC development, suggesting that it is important for tumor development [24], but to a lesser extent than in older GC patients [38]. Moreover, Rugge, et al. confirmed that *H. pylori* infection was significantly associated with both diffuse and intestinal histotypes [39]. In this sense, the low prevalence of *H. pylori* in our cohort, and the lack of differences in *H. pylori* status between histotypes, suggest that the implication of this infection in the carcinogenesis process is less relevant in EOGC, although prospective and large cohorts are needed to deepen in this observation. 

The main strength of our study is that it describes not only clinical but also histological and molecular data of a large cohort of more than 300 patients with EOGC, although we are aware of the limitations associated to an observational and retrospective study.

## 5. Conclusions

Our results show that that most early onset GC cases are diagnosed in advanced stage, have diffuse histology, and have infrequent DNA mismatch repair somatic deficiency. Moreover, early onset GC has a marked genetic heterogeneity. Thus, it is essential to consider familial history of tumors, not only GC, but also and more importantly other tumors related with hereditary syndromes (such as colorectal, breast, and ovarian cancer), in order to select adequate patients to perform a suitable genetic counseling and enhance the emerging use of multigene panels. 

## Figures and Tables

**Table 1:** Clinical characteristics of the patients (N = 309).

	EOGC
Age (years) at diagnosis; median (IQR)	44 (40–48)
Gender, number (%): Man	191 (61.8)
**Environmental risk factors; number (%)**	
*Helicobacter pylori* infection	24/82 (29.3)
Smokers	77/169 (45.6)
Moderate or high alcohol consumers	21/105 (20)
**Personal history of extra-gastric cancer (*n* = 309); number (%)**	
Breast and/or ovarian	5 (1.6)
Lung	1 (0.3)
Thyroid	1 (0.3)
Hodgkin lymphoma	1 (0.3)
Cervix	1 (0.3)
**Familial history of cancer (*n* = 117); number (%)**	
GC	18 (15.4)
Colorectal	8 (6.8)
Ovarian and/or breast	15 (12.8)
Others	39 (33.3)
Criteria of familial GC	5 (4.3)

IQR, interquartile range; EOGC, early onset gastric cancer; GC, gastric cancer.

**Table 2:** Characteristics of the tumors (*N* = 309).

	EOGC
**Gastric location (N = 203); number (%)**	
Cardias	3 (1.5)
Fundus	18 (8.9)
Body	111 (54.7)
Antrum	50 (24.6)
Extensive	21 (10.3)
**Stage (N = 212); number (%)**	
I/II	44 (20.8)
III/IV	166 (78.3)
**Histology (N = 161); number (%)**	
(a) Diffuse Signet ring cell subset	118 (73.3) 38/118 (32.2)
(b) Intestinal	30 (18.6)
(c) Mixed	13 (8.1)
Tumor differentiation grade (N = 23/43) number (%) *	
High grade (poorly differentiated)	9 (39.1)
Low-grade (well/moderately differentiated)	14 (60.9)
**Treatment (N = 205) number (%)**	
Surgery	40 (19.5)
Surgery + chemotherapy	58 (31.2)
Chemotherapy	64 (28.3)
Surgery + chemotherapy + radiotherapy	24 (11.7)
Chemotherapy + radiotherapy	3 (1.5)
Palliative	16 (7.8)

EOGC, early onset gastric cancer. * According to the WHO classification (2019), the degree of differentiation only applies to the intestinal type.

**Table 3:** Characteristics in patients with hereditary syndromes.

Patient	Age	Gender	Tumor Characteristics	*H. pylori* Infection	Personal History of Other Tumors	Familial History of GC	Familial History of Other Tumors	MMR-IHC	Gene (Pathogenic Variant)
1	49	Woman	Intestinal hist. Stage II	Not available	Breast	No	Ovarian and breast	MMR+ (normal)	*BRCA2* (c.3166C>T; p.Gln1056 *; nonsense)
2	38	Man	Diffuse hist. Multifocal (plastic linitis) Stage IV	No	No	Yes	No	MMR+ (normal)	*CDH1* (c.2164+5G>C; splicing)
3	34	Man	Diffuse hist. Multifocal (plastic linitis) Stage IV	No	No	No	Breast and colorectal	MMR+ (normal)	*TP53* (c.365_366delTG; p.Val122fs; frameshift)
4	40	Man	Diffuse hist. Multifocal (plastic linitis) Stage IV	No	No	Yes	No	MMR+ (normal)	*CDH1* (c.187C>T; p.Arg63 *; nonsense)

hist., histology; GC, gastric cancer; MMR-IHC, immunohistochemistry of DNA mismatch repair proteins; MMR, tumor mismatch repair; *, stop codon

## Data Availability

The data presented in this study are available on request from the corresponding author.

## Supplementary Materials

The following are available online at https://www.mdpi.com/article/10.3390/cancers13133132/s1, Figure S1: Example of visualization using Integrative Genomics Viewer of two of the germline variants identified. (a) *CDH1* c.2164+5G>C (splicing variant); (b) *TP53* c.365_366delTG (p.Val122fs; frameshift variant).
