# Supplementary material for: Clinical, Molecular and Genetic Characteristics of Early Onset Gastric Cancer: Analysis of a Large Multicenter Study
Source: Cancers (Basel). 2021 Jun 23;13(13):3132. doi: 10.3390/cancers13133132 (PMC8269053; doi:10.3390/cancers13133132)
Supplement: Supplementary file 1 [file cancers-13-03132-s001.zip › cancers-1167340-supplementary.pdf]

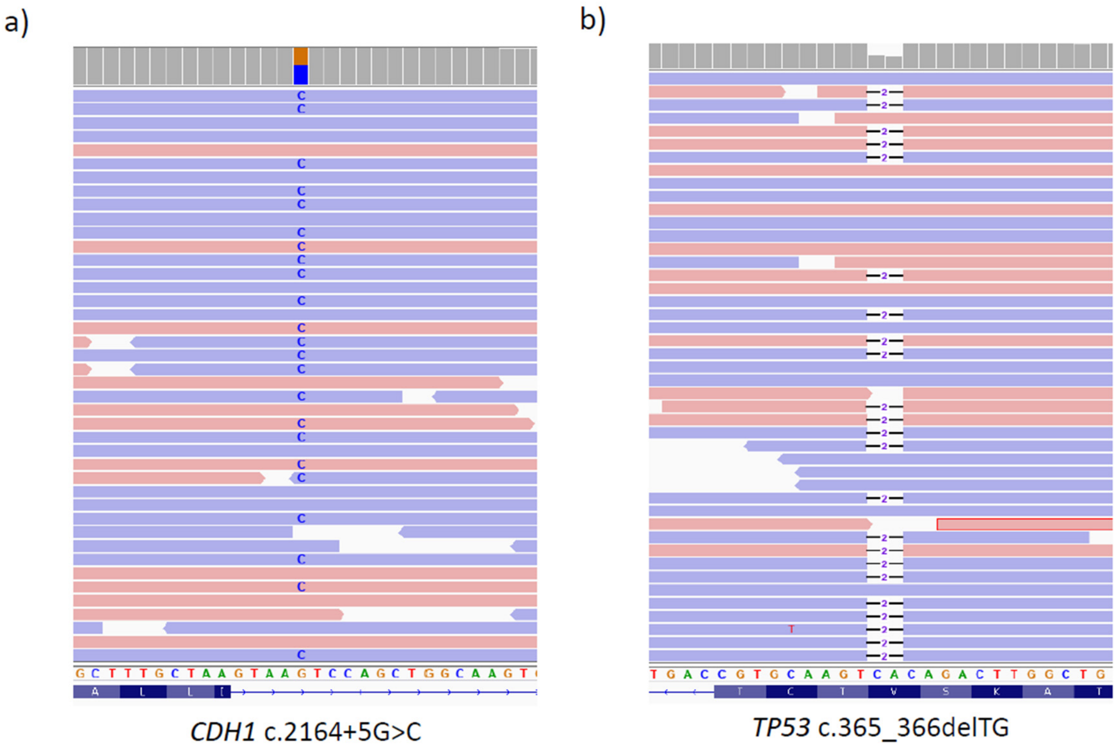

Figure S1: Example of visualization using Integrative Genomics Viewer of two of the germline variants identified. (**a**) *CDH1* c.2164+5G>C (splicing variant); (**b**) *TP53* c.365_366delTG (p.Val122fs; frameshift variant).
